# A Novel Hepatitis C Virus Genotyping Method Based on Liquid Microarray

**DOI:** 10.1371/journal.pone.0012822

**Published:** 2010-09-20

**Authors:** Cesar A. B. Duarte, Leonardo Foti, Sueli M. Nakatani, Irina N. Riediger, Celina O. Poersch, Daniela P. Pavoni, Marco A. Krieger

**Affiliations:** 1 Instituto Carlos Chagas-Fundação Oswaldo Cruz (FIOCRUZ), Curitiba, Brazil; 2 Instituto de Biologia Molecular do Paraná (IBMP), Curitiba, Brazil; 3 Laboratório Central do Estado (LACEN-PR), São José dos Pinhais, Brazil; 4 Department of Gastroenterology, School of Medicine, University of São Paulo (USP), São Paulo, Brazil; Tsinghua University, China

## Abstract

The strategy used to treat HCV infection depends on the genotype involved. An accurate and reliable genotyping method is therefore of paramount importance. We describe here, for the first time, the use of a liquid microarray for HCV genotyping. This liquid microarray is based on the 5′UTR — the most highly conserved region of HCV — and the variable region NS5B sequence. The simultaneous genotyping of two regions can be used to confirm findings and should detect inter-genotypic recombination. Plasma samples from 78 patients infected with viruses with genotypes and subtypes determined in the Versant™ HCV Genotype Assay LiPA (version I; Siemens Medical Solutions, Diagnostics Division, Fernwald, Germany) were tested with our new liquid microarray method. This method successfully determined the genotypes of 74 of the 78 samples previously genotyped in the Versant™ HCV Genotype Assay LiPA (74/78, 95%). The concordance between the two methods was 100% for genotype determination (74/74). At the subtype level, all 3a and 2b samples gave identical results with both methods (17/17 and 7/7, respectively). Two 2c samples were correctly identified by microarray, but could only be determined to the genotype level with the Versant™ HCV assay. Genotype “1” subtypes (1a and 1b) were correctly identified by the Versant™ HCV assay and the microarray in 68% and 40% of cases, respectively. No genotype discordance was found for any sample. HCV was successfully genotyped with both methods, and this is of prime importance for treatment planning. Liquid microarray assays may therefore be added to the list of methods suitable for HCV genotyping. It provides comparable results and may readily be adapted for the detection of other viruses frequently co-infecting HCV patients. Liquid array technology is thus a reliable and promising platform for HCV genotyping.

## Introduction

Hepatitis C virus (HCV) infection occurs worldwide and it has been estimated that 180 million people are infected globally [1]. This infection is a common cause of chronic liver disease and the most common indication for liver transplantation [2], [3]. Treatment should be tailored according to the genotype of the virus. Patients with genotype 1 viruses should be treated for 48 weeks with peginterferon alfa-2a plus standard weight-based ribavirin, whereas patients with viruses of genotypes 2 and 3 should be treated with peginterferon alfa-2a plus low-dose ribavirin for 24 weeks [4]. The most strongly conserved regions of the HCV genome are the 5′ untranslated region (5′UTR), and the terminal 99 bases of the 3′ untranslated region [5]. The diagnostic assays based on the 5′UTR currently used have an acceptable accuracy, displaying more than 95% concordance with the results of genotyping based on sequencing of the NS5B polymerase gene in the variable region. The results of the sequencing of this region are consistent with those of whole-genome sequencing for genotype/subtype determination [6]. Two commercial assays are frequently used to determine HCV genotypes: the TruGene 5′NC HCV Genotyping kit (Siemens Healthcare Diagnostics Division, Tarrytown, NY), based on direct sequence analysis of the 5′UTR (untranslated region), and the Versant™ HCV Genotype Assay LiPA (version I; Siemens Medical Solutions, Diagnostics Division, Fernwald, Germany), based on reverse hybridization analysis with genotype-specific oligonucleotide probes binding to the 5′UTR [7]. Given the importance of HCV genotyping, an accurate, sensitive, cost-effective and reproducible assay is much needed. Ideally, any diagnostic method for HCV genotyping should be able to distinguish between all the relevant variants simultaneously. We describe here, for the first time, an HCV genotyping method based on liquid microarray technology (xMAP Technology, Luminex Corp, Austin, Texas).

Liquid microarray technology has been used for the detection of many different pathogens [8], [9], [10]. One of its key properties is an extensive multiplexing capacity, making it possible to detect different nucleic acid targets simultaneously. This is particularly important for HCV genotyping, as there are many different HCV genotypes and subtypes [11]. HCV genotyping on the basis of two different viral genome regions has the advantage that the results for one region confirm those for the other, and should also make it possible to detect rare cases of inter-genotypic recombination [12], [13].

In this study, we developed an innovative new method for HCV genotyping based on the simultaneous analysis of two genomic regions, the 5′UTR and NS5B regions, (nonstructural region 5B), for all samples tested. The analysis of two regions provides a means of confirming the results obtained and could potentially detect inter-genotypic recombination. The liquid microarray HCV genotyping test performed as well as the test currently used (Versant™ HCV genotype Assay LiPA). It also has the advantage of being less subjective as it does not rely on visual reading as LiPA, but Luminex software generated numerical value (MFI) [6], [14]. Visual reading interpretation may be subjected to inter- operators' variation and possible misinterpretation [15].

## Results

**Figure 1:**
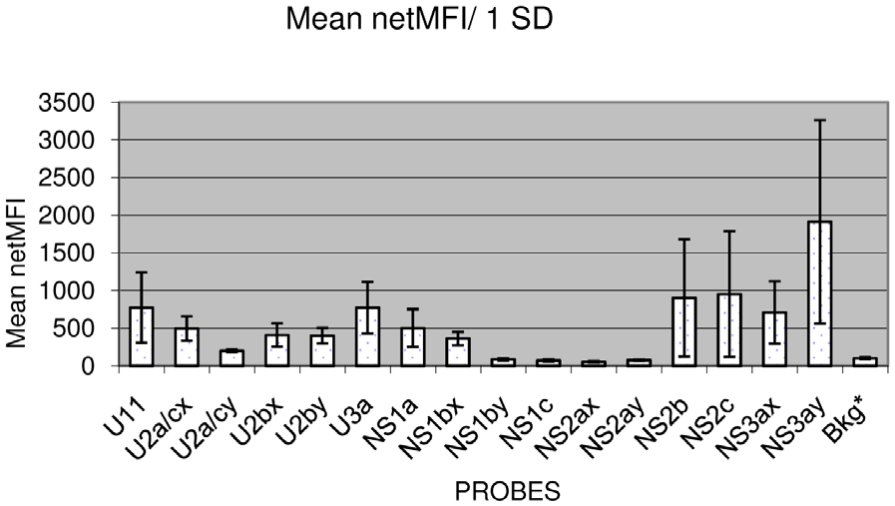
Mean netMFI and standard deviation (SD). MFI: Median fluorescence intensity; netMFI: MFI - mean background (blank) MFI.*Bkg =  background. Hybridization signals above background were not detected for probes NS1by, NS1c, NS2ax and NS2ay.

All 78 samples were tested in duplicate, from RNA extraction and RT-PCR to hybridization and Luminex reading. According to the criteria outlined in the materials and methods, 74 samples generated a specific signal for at least one microsphere/probe set. All probes binding to region 5′UTR and six of the 10 probes binding to NS5B generated hybridization signals with various samples (figure 1). The netMFI values obtained for all samples are shown in the supporting information (table S1). The hybridization of PCR products to specific 5′UTR and NS5B probes attached to microspheres resulted in different netMFI profiles. Genotype 1 samples invariably gave a hybridization signal for probe U11 (5′UTR region). However, for genotype 1 strains also giving hybridization signals with NS5B probes, subtypes were identified as 1a (signal for probe NS1a) or 1b (signal for probe NS1bx). Samples identified as subtype 2b displayed one of the following three combinations: signal for the U2bx probe only, signals for U2bx + NS2b or signals for U2By + NS2b. Samples identified was subtype 2c hybridized with the U2a/cx + U2a/cy + NS2c probes or the U2a/cx + NS2c probes. One sample giving a signal only with the U2a/cx probe was classified as subtype 2a or 2c. Samples identified as subtype 3a displayed one of the following four combinations: signal for U3a1 only, signals for U3a1 + NS3ax, signals for U3a1 + NS3ay or signals for U3a1 + NS3ax + NS3ay (table 1).

**Table 1:** Hybridization signal profiles.

Samples	Genotype^1^	U11	U2a/cx	U2a/cy	U2bx	U2by	U3a	NS1a	NS1bx	NS1by	NS1c	NS2ax	NS2ay	NS2b	NS2c	NS3ax	NS3ay
28	1	+	-	-	-	-	-	-	-	-	-	-	-	-	-	-	-
12	1a	+	-	-	-	-	-	+	-	-	-	-	-	-	-	-	-
7	1b	+	-	-	-	-	-	-	+	-	-	-	-	-	-	-	-
1	2a or 2c	-	+	+	-	-	-	-	-	-	-	-	-	-	-	-	-
1	2b	-	-	-	+	-	-	-	-	-	-	-	-	+	-	-	-
3	2b	-	-	-	+	-	-	-	-	-	-	-	-	+	-	-	-
3	2b	-	-	-	+	-	-	-	-	-	-	-	-	-	-	-	-
1	2c	-	+	+	-	-	-	-	-	-	-	-	-	-	+	-	-
1	2c	-	+	-	-	-	-	-	-	-	-	-	-	-	+	-	-
2	3a	-	-	-	-	-	+	-	-	-	-	-	-	-	-	+	+
4	3a	-	-	-	-	-	+	-	-	-	-	-	-	-	-	+	-
3	3a	-	-	-	-	-	+	-	-	-	-	-	-	-	-	-	+
8	3a	-	-	-	-	-	+	-	-	-	-	-	-	-	-	-	-

1 Genotype/subtype obtained by microarray analysis.

1, 1a, 1b, 2a, 2b, 2c and 3a: HCV genotypes/subtypes.

5′UTR and NS5B probes specificity - U11: Genotype 1; U2a/cx and U2a/cy: Subtypes 2a and 2c;

U2bx and U2by: Subtype 2b; U3a: Subtype 3a; NS1a: Subtype 1a; NS1bx and NS1by: Subtype 1b; NS1c: Subtype 1c;

NS2ax and NS2ay: Subtype 2a; NS2b: Subtype 2b; NS2c: Subtype 2c; NS3ax and NS3ay: Subtype 3a.

(+): Specific hybridization signal.

(-): No specific hybridization signal.

Microarray genotype determination was not possible for four samples subtyped by the Versant™ HCV assay as 1a, 1b, 2 and 3a. A comparison of the genotype/subtype results obtained with the two methods is shown in the table 2.

**Table 2:** Comparison of the results obtained with the microarray and Versant™ HCV.

Samples	Microarray	Versant™ HCV
23	1	1b
5	1	1
3	1a	1
9	1a	1a
7	1b	1
1	2a or 2c	2
7	2b	2b
2	2c	2
17	3a	3a

Samples: number of genotype and/or subtype agreement between tests.

### Clinical sensitivity

Seventy-four of the 78 samples previously tested by Versant™, were successfully genotyped by the liquid microarray method (74/78, 95%). The concordance between the two methods for these 74 samples was 100% for genotype. The following genotypes were identified: genotype 1 (47 samples), genotype 2 (10 samples) and genotype 3 (17 samples). At subtype level, the concordance between the two methods was 100% for subtypes 3a (17/17) and 2b (7/7). One samples was identified as 2a or 2c (2a by sequencing) and two samples were identified as 2c by liquid microarray analysis, confirmed by sequencing, whereas they were identified only to genotype level by Versant™. Nine of the 12 subtype 1a identifications and one of the seven subtype 1b identifications were consistent with the results of Versant™ analysis. The remaining samples were identified only to the genotype level, as genotype “1”, by Versant™. No PCR products for the NS5B region were obtained for 28 genotype 1 samples for which subtype was not determined.

### Specificity

We evaluated the rate of false positive detection with the genotype-specific primer-probe sets, by testing thirty-eight of the positive specimens for the following viruses: hepatitis B virus (n = 12) and hepatitis A virus (n = 7), for which tests are often carried out in suspected cases of HCV infection; HIV (n = 12), which was selected for study due to the high incidence of HIV-HCV co-infection in the population studied and dengue virus (n = 7), which belongs to the same family (Flaviviridae) as HCV. The genotype primer-probe sets were found to be specific for the various genotypes. They gave no false positive results with other common hepatitis viruses (HAV and HBV), dengue virus or HIV. Furthermore, two negative control samples testing negative with the Versant™ HCV assay, also tested negative with the microarray test.

## Discussion

We describe here an HCV genotyping assay based on liquid microarray technology and including 16 hybridization probes specific for different HCV genotypes and subtypes and one set of primers for each of the two regions most frequently used for HCV detection and genotyping (5′UTR and NS5B).

The liquid microarray assay described here had excellent clinical sensitivity (95%) for genotype determination. RNA instability and secondary structures have made it difficult to develop molecular tests for HCV. The viruses circulating in Brazil are almost exclusively of genotypes 1, 2 and 3 [16] and the microarray test would fulfill the needs of this large country for HCV genotyping. The clinical sensitivity obtained for this test is similar with published findings for other tests [17]. Only samples previously tested with the Versant™ HCV assay were available for microarray testing. No samples for which Versant™ HCV had failed to assign a genotype were tested with the microarray. A study including parallel sample testing would provide unbiased conclusions regarding clinical sensitivity.

Versant™ HCV Genotype Assay LiPA is by far the most used test in Brazil for HCV genotyping; its high sensitivity and specificity (close to 99%) has made it a reference standard in previous studies [6], [18]. Sample selection was based on HCV genotype as had been previously assigned using the LiPA. Performance was evaluated by comparing the genotyping results obtained from microarray with those generated by LiPA.

No PCR product for the targeted regions was obtained for four samples. This failure to generate a PCR product made genotyping impossible for these samples. Possible reasons for this lack of a PCR product include low viral titers for these samples and the inefficiency of PCR due to the presence of an inhibitor with no apparent effect on the LiPA assay.

As shown in table 3, some genotypes/subtypes were detected with more than one probe, due to the level of nucleotide variability even within subtypes. For instance, samples identified as subtype 3a, were correctly classified as such based on four different possible hybridization signal outcomes, as described in the results. In the particular case of subtype 3a, 5′UTR information alone is sufficient to assign a subtype. We were therefore able to classify samples with a hybridization signal only for the 5′UTR probe U3a in addition to those that also gave signals with probes designed to bind to the NS5B region (NS3ax, NS3ay or both).

**Table 3:** Primers and probes for the 5′UTR and NS5B regions.

Oligonucleotide	Genomic region	Genotype/subtype	Modification	Sequence 5′→3′	Positioning*
HCV1F	5′UTR		biotin	CGGGAGAGCCATAGTGGT	130–147
NSNOF	NS5B		biotin	ACMAAGCYCAAACTCACTCCAWT	1597–1621
HCVRX	5′UTR			CGCRACCCAACRCTACT	256–273
NSNOR	NS5B			CAYGMGACACGCTGTGA	1685–1702
U11	5′UTR		amino-12C	ACCCGGTCGTCCTGGCAATT	179–198
U3a	5′UTR	3a	amino-12C	ACCCGGTCACCCCAGCGATT	179–198
U2a/cx	5′UTR	2a & 2c	amino-12C	ACCCAGTCTTCCCGGCAATT	179–198
U2a/cy	5′UTR	2a & 2c	amino-12C	ACCCGGTCTTCCCGGCAATT	179–198
U2bx	5′UTR	2b	amino-12C	ACCCAGTCTTTCCGGTAATT	179–198
U2by	5′UTR	2b	amino-12C	ACCCAGTCTTTCCGGCAATT	179–198
NS1a	NS5B	1a	amino-12C	GTAGCCAGCCGTGAACCA	1652–1668
NS1bx	NS5B	1b	amino-12C	GTAACCAGCAACGAACCA	1652–1668
NS1by	NS5B	1b	amino-12C	GTAGCCAGCAATGAACCA	1652–1668
NS1c	NS5B	1c	amino-12C	GTAACCGCCCGTGAACCA	1652–1668
NS2ax	NS5B	2a	amino-12C	GGCGCCGACAGTGAACCA	1652–1668
NS2ay	NS5B	2a	amino-12C	GGCGCCGACGGTAAACCA	1652–1668
NS2b	NS5B	2b	amino-12C	GGCGCCCACGGTGAACCA	1652–1668
NS2c	NS5B	2c	amino-12C	AGCGCTGACGGTGAACCA	1652–1668
NS3ax	NS5B	3a	amino-12C	GACACCAACCGTAAACCA	1652–1668
NS3ay	NS5B	3a	amino-12C	GACGCCAACCGTAAACCA	1652–1668

*Reference sequence (GenBank, AF009606). M: A or C; Y: C or T; W: A or T; R: A or G.

5′UTR (untranslated region) specific primers: HCV1F (forward), HCVRX (reverse).

NS5B (nonstructural 5B region) specific primers: NSNOF (forward), NSNOR (reverse).

Modification: Forward primer had 5′ biotin added for streptavidin-R-phycoerythrin (SA-PE) ligation.

Probes had 5′ amino-12C (carbon linker) added for ligation to the microspheres.

By contrast, hybridization patterns with the 5′UTR probe are not sufficiently informative for the subtyping of genotype 1 and 2 viruses. For these genotypes, information concerning the NS5B region is required for the accurate determination of subtype. No 1c subtype virus was identified with the Versant™ HCV assay and, consistent with this finding, no signal was obtained with the NS1c probe.

Information about the 5′UTR was sufficient to assign samples to subtype 2b, but not to differentiate between subtypes 2a and 2c. Differentiation between these two subtypes required hybridization signals for probes binding to the NS5B region. Two samples were classified as subtype 2c because they gave a hybridization signal with the NS2c probe, and one sample was identified as subtype “2a or 2c” on the basis of the signal obtained for the U2a/cx probe, which hybridizes to both subtypes, and the absence of a signal for probes binding to the NS5B region.

Some cross-hybridization was observed between three of the 10 genotype 2 samples, with 5′UTR probes only. The *gen997*, *gen1859* and *genat* samples were identified as belonging to subtypes “2a or 2c”, 2b and 2c, respectively. For *gen997* and *genat* the netMFI obtained with the U2a/cx probe was more than twice that obtained with the U2by probe (supporting information, table S1). The opposite pattern was observed with *gen1859,* the netMFI for the U2by probe being more than four times that for the 2a/cx probe. This cross-hybridization was attributed to the high similarity between the probes (table 3). Nonetheless, probes were designed to bind at the same positions, and any mismatch decreases binding to the corresponding probe, thereby reducing the resulting netMFI to levels lower than those for perfect hybridization in a situation of competitive dynamics. This is why it was possible to determine genotype/subtype in these samples, even in the presence of some cross-hybridization.

The lower efficiency of determination for subtypes 1a/1b was due to the failure of multiplex PCR to amplify the NS5B region in a significant number of the samples tested. This failure of multiplex PCR may result from the high nucleotide sequence diversity of the NS5B region, impairing primer annealing. Subtype determination based on the 5′UTR region was possible in all samples for subtypes 2b, and 3a, but it has been demonstrated that the 5′UTR region alone is not sufficiently accurate to distinguish between genotypes 1a and 1b in many cases [19], [20]. Further improvements in PCR amplification of the NS5B region are required, to increase the efficiency of subtype discrimination. This could be accomplished by decreasing the degeneracy of the primer set for the amplification of the NS5B region. Two separate PCRs for the NS5B region, for genotype 1 and genotype 2, could be carried out in parallel.

Even though 1a/1b subtype determination in this assay was below of expected, it is worth considering that subtyping is irrelevant as a consideration for therapeutic strategy, being of only epidemiological importance [4]. The microarray assay was able of consistently determining genotype which matters on treatment planning and was the main goal of this study.

Furthermore, the microarray format potentially can be adapted to cover other genotypes prevailing in other geographic areas as it has been conceived to permit inclusion of probes up to the theoretical number of one hundred [14].

This is facilitated by the presence of TMAC in the buffer, which homogenizes the hybridization temperatures of different probes by stabilizing A/T pairing. The hybridization temperature used is therefore much more dependent on the extension function of the probe than on its nucleotide composition [21].

Inter-genotypic recombination detection is rare, but an assay with the capacity to detect it could prevent inappropriate treatment. This is particularly important for tests based on the 5′UTR region. For example, genotyping based on the 5′UTR region of a recombinant strain would lead to treatment for the genotype detected at that site, but the virus might have the NS5 region of another genotype, and this second region is known to be associated with IFN susceptibility [22]. All of this is hypothetical, and further, well designed studies would be required for confirmation. No evidence of inter-genotypic recombination was found among the 74 samples tested, despite the assay having been designed to detect such recombination. However, this lack of detection may be due to the small number of samples, as inter-genotypic recombination is a rare event.

Despite the small number of samples evaluated for specificity, we detected no non-specific hybridization of the probes used to non-HCV targets. This aspect is particularly important, given the high prevalence of HIV co-infection in HCV patients and the risk factors common to these two types of infection.

The total time required for performing this assay was three and a half hours, virtually the same as required for LiPA v. 1. The estimated reaction cost of microarray assay was eight times lower than that of available commercial methods in Brazil. In order to estimate the cost of the microarray assay, costs of NucliSens EasyMAG extraction kit (bioMerieux), Omniscript Reverse Transcriptase (Qiagen®), *Taq* polymerase®, primers and probe sets (synthesized by Invitrogen), RNase inhibitor (Invitrogen), and Luminex beads and reagents were taken for comparison.

Assessment of the final costs related to LiPA v.1 included the Amplicor® Hepatitis C Virus-HCV-Test, version 2.0 Roche (for PCR product generation) and the VERSANT™ HCV Genotype Assay-LiPA v.1 (Siemens). Tests costs estimation was based on the currently available commercial reagent prices in Brazil at the time the study was conducted. Equipment maintenance, human resources and other indirect costs were not taken into account for comparison and calculation. The reduction costs associated with this new assay would make possible to use it in all Brazil regions, many of which still does not have HCV genotyping capacity, considerably impairing treatment strategy.

When choosing a molecular diagnostic method, it is also important to take into account the hands-on time and throughput potential. All the steps of microarray assays can be automated, overcoming the need for interpretation by highly skilled staff and the possible errors associated with handling [23], [24], [25].

In conclusion, this new xMAP Luminex assay system is rapid, sensitive, and specific for the purposes of HCV genotyping. It can be used for the simultaneous detection of different HCV genotypes and subtypes. This system is highly flexible, allowing the inclusion of new sequences, such as genetic variants, or additional viruses, by the simple addition of appropriate microsphere sets to the multiplexed mixture. Liquid microarray technology should, in the near future, become widely used in nucleic acid detection.

## Materials and Methods

### Samples

Plasma was obtained from blood samples collected into ethylenediaminetetraacetic acid (EDTA) between January 2007 and February 2008. These samples were obtained from 78 patients with chronic HCV infection at the Paraná State Reference Laboratory of Health (LACEN-PR). Plasma was stored at -70 °C until use. The HCV genotypes for these samples had previously been determined with the Versant™ HCV genotype Assay LiPA (version I; Siemens Medical Solutions, Diagnostics Division, Fernwald, Germany). The sequences obtained have been submitted to GenBank and can be retrieved under accession numbers FJ159697 to FJ159843.

The study protocol was approved by the Ethics Committee of University of São Paulo (CAAE -2546.0.015.000-05). The requirement for written informed consent was waived by the IRB because the samples used were coded upon collection and storage. Personal identifiers have been removed, avoiding patient identification completely. The samples cannot be traced to individual patients. Besides, the study did not present any risks for the patients and involved no procedures for which written consent is normally required outside of the research context.

### RNA extraction

RNA was extracted from 200 µl of EDTA-treated plasma from each patient sample, with the Nuclisens easyMAG automated platform (bioMerieux, Inc., Boxtel, Netherlands), used according to the manufacturer's instructions. Briefly, 200 µl of an EDTA-treated plasma sample was added to 2 ml of lysis buffer and the mixture was incubated for 10 min at room temperature. The lysed sample was then transferred to the well of a plastic vessel containing 100 µl of magnetic silica. Automatic magnetic separation was then carried out and nucleic acid was recovered in 20 µl elution buffer.

### Primers and probe design

All the probes and primers used in this assay were designed on the basis of consensus sequences deposited at Los Alamos National Laboratory (Hepatitis C Virus Database Project) http://hcv.lanl.gov/components/hcv-b/GET_ALIGNMENTS/get_alignment.comp. Two sets of primers were designed: one set for the 5′UTR region and the other for the NS5B region. The forward primers were 5′ biotinylated. The oligonucleotides (probes) to be attached to the microspheres were designed so as to contain a 5′ amino 12-carbon linker. These probes were designed to be complementary to the internal portion of the biotinylated strand from PCR products: six probes for the 5′UTR region and 10 for the NS5B region (table 3).

### Reverse transcription and PCR

We carried out 88 reverse transcription reactions (78 HCV samples and 10 negative controls) with the Omniscript® Reverse Transcription Kit (Qiagen). Targets were reverse transcribed in 20 µl reaction mixtures containing 10× RT buffer, 10 units RNase inhibitor, 4 units Omniscript Reverse Transcriptase, 4 µg random primers, 0.5 mM of each dNTP and 12.5 µl RNA, for one hour at 37°C. A multiplex PCR was then carried out in 10× PCR buffer, 0.3 units *Taq* polymerase, 1.5 mM MgCl_2_, 200 µM of each dNTP, 0.2 µM each of the HCVF/HCVRX primers and 0.5 µM each of the NSNOF/NSNOR primers. PCR was conducted in a Peltier Thermal Cycler (MJ96+/MJ966), by incubation at 94°C for 5 min, followed by 35 cycles of denaturation at 94°C for 30 s, annealing at 56.8°C for 30 s, and extension at 72°C for 60 s. There was then a final extension phase at 72°C for 3 min, and reactions were held at 4°C thereafter.

### Attachment of probes to microspheres

Probes, purchased with a 5′ amino 12-carbon linker (The Midland Certified Reagent Company, Midland, TX), were covalently coupled to carboxylated microspheres (Luminex Corp., Austin, TX), using a carbodiimide coupling procedure. Carboxylated microspheres were prepared by placing 200 µl of stock suspension in a 1.5 ml microcentrifuge tube and centrifuging at 8,000 g for 2 min. The supernatant (storage buffer) was decanted, and the microspheres were resuspended in 50 µl of 0.1 M MES [2-(N-morpholino) ethanesulfonic acid], pH 4.5. Microspheres were exposed to light as little as possible to prevent photobleaching, which prevents the Luminex detection system from registering the individual microsphere codes. Once suspended, the microspheres were vortexed and 2 µl of 100 µM amino-modified probe suspension was added. Aliquots of desiccated 1-ethyl-3-(dimethylaminopropyl) carbodiimide HCl (EDC) powder (Pierce Biotechnology Rockford, IL, USA) were warmed to room temperature. We added 2.5 µl of 10 mg/ml EDC to each mixture, for the attachment of the amine-modified probes to the carboxylated microspheres. The microsphere mixture was incubated in the dark at room temperature for 30 min. A second aliquot of 10 mg/ml EDC was then added and the suspension incubated for another 30 min. After incubation with EDC, 1 ml of 0.02% polyoxyethylenesorbitan monolaurate (Tween 20) was added and the microspheres were vortexed and then centrifuged at 8,000 g for 2 min. The supernatant was removed by aspiration. We then added 1 ml of 0.1% sodium dodecyl sulfate. The microspheres were vortexed and then centrifuged again at 8,000 g for 2 min. The supernatant was once again removed by aspiration. The microspheres were then resuspended in TE buffer (10 mM Tris-HCl, 1 mM EDTA, pH 8.0) and the number of microspheres per microliter was determined with a hemacytometer. Sets of microspheres with the newly attached probes were then stored at 4°C for future use.

### Hybridization with PCR products

The hybridization assay was based on the binding of complementary biotinylated PCR products to the probes attached to the microspheres. The assay was performed in a 96-well, clear, low-profile, flat-top PCR microplate (Axygen Inc). The final reaction volume was 50 µl, including 33 µl of microsphere mixture and 17 µl of the amplified product or TE buffer (blank). The microsphere mixture was prepared by adding a calculated volume of each microsphere set to 1.5× TMAC buffer (1.5× TMAC buffer is 4.5 M tetramethylammonium chloride, 75 mM Tris-HCl pH 8.0, 6 mM EDTA and 0.15% Sarkosyl), to achieve a concentration of 2,500 microspheres per set in 33 µl. We added 5 µl of PCR product; the titer plate was sealed and the amplified DNA was denatured at 95°C for 5 min and then incubated for 30 min at 55°C in a thermocycler (Peltier Thermal Cycler, MJ96+/MJ966). We then added 25 µl of a 15 µg/ml dilution of streptavidin-R-phycoerythrin (SA-PE) (Molecular Probes, Eugene, OR). The plate was incubated for 15 min at 55°C and read on a heated stage at the same temperature as the Luminex 100 platform. This platform analyzes polystyrene microspheres of 5.6 µm that are internally dyed with two different fluorochromes mixed in different ratios to generate microsphere populations with specific spectral addresses. These microspheres are classified by two lasers, a 635-nm red diode laser to detect the internal dyes of the microspheres and a 532-nm laser that excites the reporter molecule, R-phycoerythrin (PE). Each sample was run in duplicate with ten blanks per plate. The median fluorescence intensity (MFI) of the SA-PE conjugate bound to 100 microspheres of each population was reported.

### Reading the MFI signal

Median fluorescence intensity (MFI) was determined for each reaction individually. The mean background (blank) MFI was then subtracted, generating netMFI. A netMFI greater than the mean background MFI plus 10 times the standard deviation (SD) was considered to indicate specific hybridization.

## Supporting Information

Table S1 NetMFI from all samples. * Samples previously genotyped by VersantTM HCV. ¤ Samples with positive net MFI signal. ¥ Samples failured to amplify NS5b region.
(0.08 MB XLS)
